# Acute health effects of ambient air pollution including ultrafine particles in a semi-experimental setting in young, healthy individuals

**DOI:** 10.1186/s12989-025-00628-7

**Published:** 2025-05-23

**Authors:** Elisabeth Folwarczny, Felix Forster, Rudolf A. Jörres, Stefan Rakete, Sheng Ye, Mark Wenig, Nadine Gawlitta, Jürgen Schnelle-Kreis, Richard Winterhalter, Alexander Müller, Dennis Nowak, Stefan Karrasch

**Affiliations:** 1 Institute and Clinic for Occupational, Social and Environmental Medicine, LMU University Hospital, LMU Munich, Munich, Germany; 2 Comprehensive Pneumology Center Munich (CPC-M), Member of the German Center for Lung Research (DZL), Munich, Germany; 3 Department of Physics, Meteorological Institute, LMU, Munich, Germany; 4 Joint Mass Spectrometry Center (JMSC) at Comprehensive Molecular Analytics (CMA), Helmholtz Zentrum München, Neuherberg, Germany; 5 Institute for Occupational Health and Product Safety, Environmental Health Protection, Bavarian Health and Food Safety Authority, Munich, Germany; 6 TUM School of Medicine and Health, Department of Clinical Medicine - Clinical Department for Internal Medicine I, University Medical Center, Technical University of Munich, Munich, Germany

**Keywords:** Air pollutants, Particulate matter, Ultrafine particles, Short-term exposure, Lung function, Lung volume measurements, Fractional exhaled nitric oxide testing

## Abstract

**Background:**

Multiple effects of ultrafine particles (UFP) on human subjects are known but there is less knowledge of how relative exposure levels between ultrafine and fine particles as typically encountered in large cities affect lung function and cardiovascular parameters.

**Methods:**

Four sites with high/low levels of ultrafine particles and/or fine particles were selected in the city of Munich, Germany: control area (woodland), urban environment, heavy traffic site, biomass combustion (beech wood). In a randomized cross-over design, 26 young, healthy individuals were exposed at each site over 75 min to atmospheric pollutants, which were monitored continuously, while performing intermittent (5 min per 15 min) light exercise. Parameters assessed pre and post exposure comprised symptoms, spirometry, lung diffusing capacity for carbon monoxide (DLCO) and nitric oxide (DLNO), alveolar volume (AV), the fractional concentration of exhaled nitric oxide (FeNO), reactive hyperemia index (RHI), blood pressure, and heart rate. Outcomes were expressed as percent changes of parameters and analyses performed by either comparing the four sites or by multiple linear regression analyses using the measured pollutant levels.

**Results:**

The sites showed the planned pattern of exposure levels but with large overlap. Outcomes showed no statistically significant differences between sites, except for symptoms which were elevated with heavy traffic site exposure and biomass combustion. In regression analyses, AV decreased by 0.92 (95% confidence interval (CI): 0.28 to 1.57) % per 10,000/cm^3^ UFP; similarly, for LDSA (lung-deposited surface area), which was highly correlated with UFP. Overall, FeNO slightly increased after exposure, but this increase was attenuated by 5.4 (95% CI: 1.8 to 9.2) % per 10 ppb ambient NO_2_. Heart rate decreased after exposures overall; this decrease was enhanced by 2.1 (95% CI: 0.3 to 4.0) % per 10,000/cm^3^ UFP.

**Conclusions:**

Short-term exposures to UFP elicited a reduction in the lung volume (AV) accessible to gas transport by diffusion and convection. FeNO was slightly elevated after all exposures, but this increase was significantly smaller at higher ambient NO_2_ concentrations. While these effects were too small to be clinically relevant, they demonstrated that typical levels of urban air pollution had measurable acute effects in young, healthy individuals.

## Introduction

Health effects of ambient air pollution, including particulate matter and various gases have been described in a multitude of studies [1–6]. These effects extend beyond the lungs, impacting other organs, particularly the cardiovascular system. It is believed that such effects are mediated not only by inflammation elicited in the lung but also by ultrafine particles (UFP) entering systemic circulation, thereby reaching various body compartments [7]. Several studies focusing on UFP suggested a specific health risk associated with such exposures [8], but epidemiological analyses were often hampered by the fact that UFP are not routinely measured, and more epidemiological studies specifically addressing this issue are needed. The lack of sufficient data was also the reason why the recommendation for air quality standards issued by the World Health Organization (WHO) in September 2021 [9] did not include specific proposals for the regulation of UFP.

Specific effects of UFP have been investigated in experimental exposure studies, particularly those using diesel exhaust [8, 10]. However, this type of study might be criticized for not accurately representing real-world exposure scenarios, which typically are characterized by a mix of air pollutants. In response, researchers have adopted semi-experimental designs, comparing exposure sites with different levels and compositions of air pollutants. An influential semi-experimental study performed in London compared health outcomes of patients between walks in Hyde Park and on Oxford Street [4]. The air pollutant levels at these two sites reflected extreme exposure differences, and clear acute effects of air pollution were observed in the elderly participants, including those with COPD or cardiac disease as well as healthy individuals. However, due to the complex mix of pollutants, the study did not allow specific conclusions regarding UFP.

This raises the question whether specific effects of UFP can be identified in study designs that better disentangle the effects of various pollutants. Since real-world exposures cover a spectrum of characteristics, this would likely require the comparison of more than two sites. Moreover, if acute effects in young healthy individuals are assessed, sensitive methods of outcome assessment are essential. If acute responses occur, they could indicate a potential for both the aggravation of pre-existing diseases and the development of new ones. Since younger individuals generally have a stronger defense capacity than older individuals, including more active and powerful antioxidant mechanisms, this approach could at least provide an estimate of the minimum health risk from ultrafine particles. To further characterize the potential impact of fine particles, LDSA was used, i.e., the lung deposited surface area of particles, which is largely due to UFP. It is thought to be a measure of the surface area of particles accumulating in the small airways and alveoli. Particles reaching this region might also enter the bloodstream [11] but LDSA emphasizes surface area. Different associations with UFP and LDSA might be informative, since number and surface area might play different roles for different outcomes.

Based on these considerations, we performed a semi-experimental exposure study with young healthy volunteers, using four exposure sites in a major city (Munich, Germany) that were selected to show different concentrations of ultrafine and fine particles, and of gases such as nitrogen dioxide (NO_2_) and ozone (O_3_), with the aim to identify specific effects of ultrafine particles in a realistic exposure setting. For this purpose, we compared the outcomes between the four selected study sites, but also included regression analyses using the actual concentrations of air pollutants assessed during each of the individual exposures.

## Materials and methods

### Measurement of ambient air pollution

For the measurement of air pollutant concentrations, mobile instruments were used. The number concentrations of ultrafine and fine particles were determined using the Condensation Particle Counter CPC 3007 (TSI Incorporated, USA; size range 0.01 to 1 μm). Concentrations of PM_2.5_ and PM_10_ were assessed using the Portable Aerosol Spectrometer (PAS) 1.108 (Grimm Technologies, Germany; size range 0.3–20 μm). Furthermore, lung density surface area (LDSA) of particles in the size range 0.01–10 μm was determined, using a multi-metric nanoparticle detector (Partector, Naneos, Switzerland). Levels of black carbon (BC) were assessed by the UV-IR Black Carbon monitor (microAeth^®^ MA200, Aethlabs, USA, flow rate 150 mL/min). NO_2_ and O_3_ were measured by using the AIR QUality Inspection boX (AIRQUIX, Meteorological Institute LMU Munich, Professor Wenig), which provided additional values for PM_2.5_ and PM_10_ that were derived from optical counts in 24 bins between 0.3 and 40 μm size. In the final analyses, the geometric mean values of the two respective measurements of PM_2.5_ and PM_10_ were used. Relative humidity and temperature were determined via a mobile weather station (WeatherScreen PRO, DNT, Germany), and the absolute water content of the air was computed, as alterations in outcomes such as FeNO might be affected by drying of the mucosa [12].

All instruments were placed on a trolley that was easy to transport and equipped with a protective cover to shield the equipment from the weather. For all exposure measures, median values were calculated, while geometric mean values were computed for PM_2.5_ and PM_10_. The median values of the 75-minute exposure periods were used in the final analyses. In the case of BC, 6 values were below the detection limit of 30 ng/m^3^ and for analysis imputed by 15 ng/m^3^.

## Selection of study sites

**Fig. 1:**
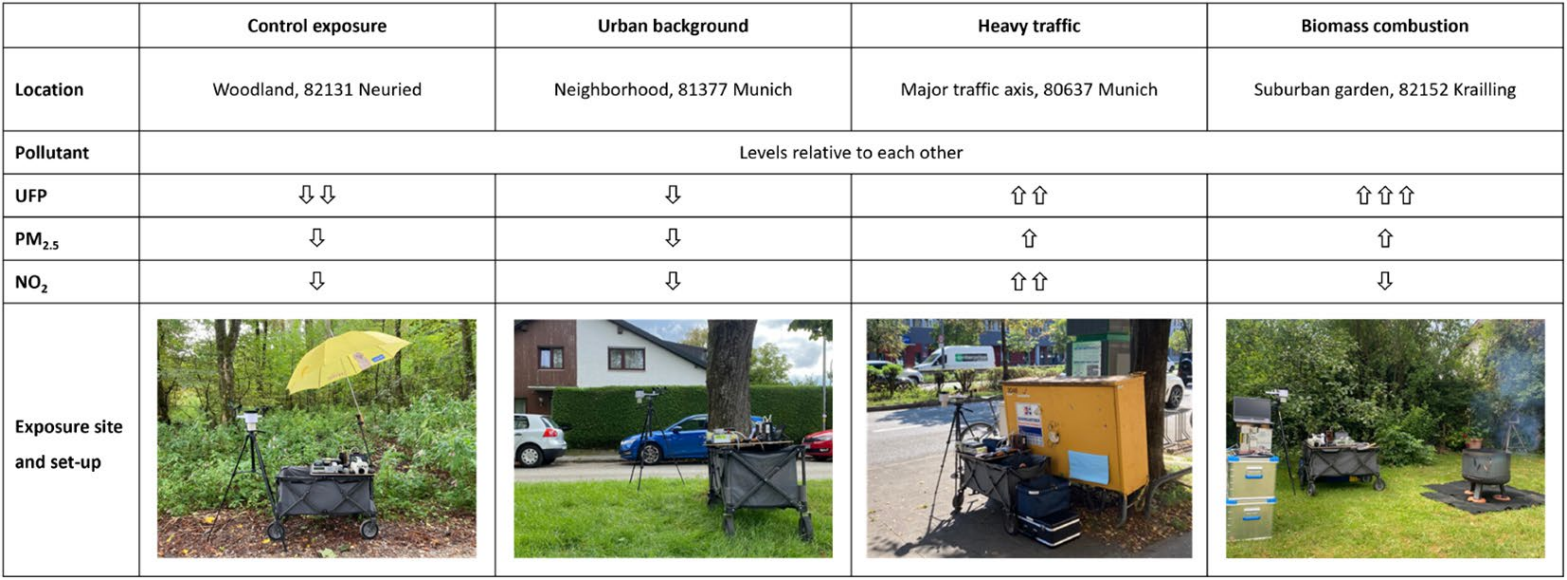
Depiction of exposure scenarios, relative exposure levels (for numerical data see Table 1) and experimental set-up. In the picture of biomass combustion, the measurement equipment from the Bavarian Health and Food Safety Authority (left side) is shown. UFP = ultrafine particles, PM = particulate matter, NO_2_ = nitrogen dioxide

We focused on identification of four types of exposures, namely “heavy traffic”, “urban background” and “control exposure” sites, and additionally “biomass combustion”. Whereas the location for “biomass combustion” was fixed due to logistic reasons, 22 locations were examined for the other sites. These were chosen to represent the most diverse and at the same time most reproducible exposure locations in the greater Munich area. The “heavy traffic” sites were in the immediate vicinity of a major road traffic axis, with high concentrations of both traffic-related UFPs and other air pollutants. For “urban background”, we examined areas as far away as possible from major traffic axes, with low concentration of UFP but possibly higher concentrations of other air pollutants. “Biomass combustion” was realized in a suburban garden by operating a fire bowl, burning beech wood, which led to high concentrations of UFP and other pollutants. For “control exposure” we tested different park areas with low concentrations of both UFP and other pollutants (see Fig. 1).

**Table 1:** Characteristics of the study participants at the screening visit. BMI = body mass index. Each of the quartiles comprised about 8 (26/4) participants

	Female	Male	Total
N	13	13	26
	**Median (1st Quartile; 3rd Quartile)[Minimum, Maximum]**		
Age (years)	27.0 (24.5; 28.5) [23, 37]	29.0 (24.0; 34.5) [22, 37]	27.5 (24.0; 30.0) [22, 37]
Height (m)	1.73 (1.67; 1.76) [1.62, 1.81]	1.85 (1.72; 1.90) [1.67, 1.93]	1.75 (1.69; 1.85) [1.62, 1.93]
Weight (kg)	66.3 (61.2; 72.3) [49.6, 87.4]	77.5 (67.7; 81.8) [51.1, 95.9]	69.4 (62.2; 80.3) [49.6, 95.9]
BMI (kg/m^2^)	22.9 (20.4; 23.7) [18.9, 28.5]	22.4 (21.8; 23.3) [18.3, 28.0]	22.4 (21.1; 23.3) [18.3, 28.5]

Based on the data, two locations each were selected for the “heavy traffic site”, “urban background” and “control exposure” locations as particularly suitable. To assess reproducibility, repeated measurements were carried out in September 2021 and between March 2022 and August 2022 at approximately the same time of the day as the planned exposures. Reproducibility was considered as important to ensure comparability between exposures across all subjects. The four final study sites were those showing the greatest differences according to the selection criteria regarding the combination of high/low UFP and high/low PM, and the highest reproducibility. Their adequacy was verified by additional measurements of particle size distributions in the range from 5 to 350 nm performed by the Bavarian Health and Food Safety Authority. During each exposure, the same equipment was used as in the selection process, to characterize each individual exposure.

### Outcome measurements

At the screening day and before exposures, the participants answered a questionnaire regarding clinical history concerning allergies, medication, infectious diseases and smoking behavior. Before and after each exposure, they answered a questionnaire covering a panel of symptoms and perceptions of exposure. For all items, the intensity was rated from 0 to 100 on a visual analogue scale (VAS), where 0 corresponded to “no symptoms” and 100 to “severe symptoms”, or “no impairment” and “strong impairment” for subjective perception. Symptoms included irritation of eyes and skin, upper and lower airways, e.g. dry cough, itchy/scratchy throat, difficulties in swallowing, cough with sputum, whistling/wheezing breathing sounds, chest tightness, respiratory distress, urge to sneeze, runny nose, nasal congestion (stuffy nose), burning sensation in nose, itchy nose, headache, feeling of dizziness, perceived cardiac/circulation disorder, nausea, burning sensation in the eyes, dry eyes, tired eyes, itchy eyes, itchy skin and skin rash/irritation. The degree of well-being, impairment of well-being, as well as disturbing smell and annoying odor during exposure was recorded only after exposures.

To assess cardiovascular effects, blood pressure at rest and heart rate were measured (boso medicus, BOSCH + SOHN, Germany), as well as endothelial function over a period of 20 min, using a finger plethysmograph (EndoPAT, Itamar Medical, Israel) yielding the reactive hyperemia index (RHI). The sequence of measurements was that outlined in the following section.

In addition, the fractional concentration of exhaled nitric oxide (FeNO) was assessed (Vivatmo Pro, Bosch Healthcare Solutions, Germany), with the aim to detect potential alterations in the surface properties of the central airways, similar to previous studies [12–14]. The concentration of exhaled carbon monoxide (eCO) was determined to confirm that subjects were non-smokers (Micro^+ TM^ Smokerlyzer, Bedfont, England). As the eCO level was elevated by the inhalation of CO during the assessment of CO diffusion capacity prior to exposure, values after exposure were also used to correct CO diffusing capacity for increased values of carboxyhemoglobin.

Lung function assessments included spirometry, in which forced expiratory volume in 1 s (FEV_1_) and forced vital capacity (FVC) were assessed (HypAir PFS, MGC Diagnostics, Belgium), as well as their ratio FEV_1_/FVC in order to detect potential airway obstruction. Moreover, the combined lung diffusion capacity for carbon monoxide (CO) and nitric oxide (NO) was determined (HypAir PFS, MGC Diagnostics, Belgium). This yielded alveolar volume (AV), transfer factors (TLCO, TLNO) for CO and NO, respectively, and the corresponding transfer coefficients (KCO, KNO) that were computed as ratio of transfer factors to alveolar volume. These indices were used to detect potential effects on the lung volume accessible to diffusion (AV) and on gas uptake, either by vascular alterations (DLCO, KCO) or alterations of diffusion characteristics (DLNO, KNO) via changes in the surface properties of the airways [12, 14, 15].

### Study participants

Participants were young, healthy adults, recruited by advertising in public areas or by direct contact, and had to meet several inclusion and exclusion criteria. Only non-smoking individuals without clinically relevant previous illnesses or perennial or seasonal allergies were eligible. Body weight and values of lung function and FeNO (fractional concentration of exhaled nitric oxide) were required to be within normal limits [16–18]. Moreover, potentially relevant exposures to airborne pollutants, due to either professional or private activities, had to be absent according to their reports.

### Study design

At a screening visit, it was assessed whether the inclusion criteria were satisfied. After giving informed consent, anthropometric data were obtained. Subjects then answered the questionnaires regarding clinical history and symptoms. Afterwards, blood pressure, FeNO and eCO were determined, followed by the determination of RHI, spirometry and combined lung diffusing capacity. Thereby, participants were able to familiarize themselves with the assessments.

Exposures followed a randomized cross-over design, with four days for each participant (one at each site). They were performed in individually randomized order and separated by at least 4 days to avoid potential bias and carry-over effects. Moreover, their time schedule was identical (11:00 a.m. to 12:15 p.m.) to account for circadian variations in physiological parameters. Exposures took place between August 2022 and May 2024, requiring average temperatures above 10 ^o^C and with breaks during wintertime. There was no instance when the ambient temperature was considered as too hot. One participant was studied per day.

The study was approved by the Institutional Research Ethics Board of the Medical Faculty of the Ludwig Maximilian University of Munich (Research Ethics Number 22–0327), and written informed consent was obtained from all participants.

### Protocol of exposure days

Participants were picked up from their homes by a commercial transport service at 08:00 a.m. and taken to the Occupational Medicine laboratory of the LMU Hospital, Campus Großhadern, Munich. The medical assessments were performed in the same order as described for the screening visit. At 10:30 a.m., transportation to the site chosen for the individual and specific day was provided. Participants wore an FFP2 mask (Aura™, 3 M Germany) to avoid effects arising from traffic exposure. After arrival, while still wearing the FFP2 mask, they waited for 10–15 min, during which the measuring system for ambient air characteristics was set up. Then, the recording of UFP, PM_2.5_, PM_10_, black carbon, NO_2_, O_3_, humidity and temperature over the whole exposure period was started.

Exposures were started by asking the participants to remove the FFP2 mask and to walk briskly over a period of 75 min. This included intermittent light exercise, as they carried a backpack weighing about 10% of their body weight for 5 min-periods, separated by 10 min-periods without backpack. All participants walked the same route at each of the four exposure sites, circling around the exposure assessment apparatus. They were instructed to walk briskly, that is, quickly but without running. Using bicycles, treadmills, or ventilation-measuring masks was not feasible due to logistical constraints. Moreover, as the exposures took place in semi-public areas, such equipment would have attracted undue attention, potentially disturbing the natural conditions of the setting. To minimize the potential influence of ambient noise, noise-reducing headphones were used. After exposures, participants put their FFP2 mask back on and were transported to the laboratory. All medical assessments performed prior to the exposure, except taking clinical history, were repeated.

### Statistical analysis

For data description, median values and quartiles, or absolute and relative frequencies were reported, depending on the type of data. We computed the relative difference (Δ%) of post-exposure vs. pre-exposure values of outcome variables, i.e., how many percentage points the post-exposure outcome was larger or smaller than the pre-exposure outcome. A Δ% of 2, therefore, means that the post-exposure outcome was 2% larger than the pre-exposure outcome, or that the post-exposure value was 102% of the pre-exposure value. Similarly, a Δ% of -2 means that the post-exposure outcome was 2% smaller than the pre-exposure outcome, or that the post-exposure value was 98% of the pre-exposure value. The final dataset contained no missing values.

To identify whether significant changes occurred, pre- and post-exposure values of each outcome were compared with each other by the nonparametric Wilcoxon matched-pairs signed-ranks test. To compare the environmental parameters between the four study sites, the nonparametric Kruskal-Wallis test was used; if there was a statistically significant overall difference, *post hoc* comparisons according to the Mann-Whitney U-test and the Benjamini-Hochberg method [19] of adjustment for multiple comparisons were performed. The percent changes were compared between the four study sites via the Friedman nonparametric test for dependent samples, with *post hoc* comparisons according to the Wilcoxon-test and again the Benjamini-Hochberg method in case that there was an overall difference. These analyses were performed using the software package SPSS (Version 29, IBM Corporation, Armonk, NJ, USA), and p values < 0.05 were considered as statistically significant.

As pollutant concentrations differed among exposure sites, but showed large heterogeneity and overlap, additionally associations of pollutant concentrations with Δ% outcome measures were analyzed. We used multilevel regression models that included a varying intercept for each individual (since each participant had 4 repeated measurements). In order to adjust UFP effect estimates for other pollutants but at the same time avoid adverse effects from multicollinearity, we included three pollutants into the same model. Since UFP and LDSA, as well as PM_2.5_ and PM_10_, as well as NO_2_ and O_3_ were highly correlated with each other, we never included these pairs together into the primary models. Instead, we investigated UFP + PM_2.5_ + NO_2_ and LDSA + PM_2.5_ + NO_2_. When O_3_ was of interest, we investigated UFP + PM_2.5_ + O_3_. All models were additionally adjusted for temperature and absolute humidity, which were included in the models in a robustly standardized form, i.e. (value - median) / median absolute deviation (a robust measure of variability). Pollutants were included in robustly centered form and divided by the magnitude of values, i.e. (value - median) / magnitude. Magnitudes were 10,000 units for UFP and 10 units for all other pollutants. Centering (value - median) has the effect that the median of the transformed variables is 0. Dividing by magnitude has the effect that effect estimates are easier to interpret. Effect estimates for UFP are, e.g., per 10,000 #/cm^3^, effect estimates for NO_2_ are per 10 ppb, etc. In two sensitivity analyses, we additionally adjusted for sex, as well as for larger pollutant sets, namely UFP + PM_2.5_ + NO_2_ + PM_10_ + O_3_ and LDSA + PM_2.5_ + NO_2_ + PM_10_ + O_3_.

Multilevel models were calculated with the Bayesian modelling platform Stan [20] using the brms package [21] in R version 4.4.1 [22]. Prior distributions were chosen to be weakly informative. The sampling procedure generated 4,000 samples (2,000 of them warm-up samples) in 4 chains each, leading to 8,000 usable samples per model. The number of samples was increased from the default 2,000 (1,000 warm-up) due to low effective sample sizes in some models. From the samples, medians and 95% posterior intervals were computed as point and interval estimates (i.e., confidence intervals; CI), respectively. After increasing the number of samples, diagnostic criteria (effective sample size, $\:\widehat{R}$, tree depth, energy, divergent transitions, and trace plots) indicated no problems during sampling [23]. For visualization of the associations, additionally quartiles of UFP, LDSA, NO_2_ and ozone levels were computed, and the percent changes of outcome measures together with their 95% confidence intervals were plotted as a function of these quartiles.

## Results

### Study participants

Initially, 45 potential participants were examined for compatibility with inclusion and exclusion criteria. Individuals with lung function values below the lower limit of normal (LLN, lower 5th percentile, *n* = 6) as defined by GLI [16, 18, 24], or other findings that might interfere with measurements (*n* = 4: high FeNO [17]; obesity, or cachexia) were excluded, while 9 subjects had to be excluded due to difficulties in scheduling the four exposure visits. Finally, 26 subjects fulfilled all criteria and were included (Table 1).

### Ambient air measurements at exposure sites

UFP number concentration in #/cm³ showed an increase from control exposure to biomass combustion, with the control exposure yielding the lowest median value of 3,162 per cm³ and biomass combustion the highest of 24,170 per cm³ (Table 2). A similar pattern was observed for LDSA. Median concentrations of 9.6 µg/m³ for PM_10_ and 4.7 µg/m³ for PM_2.5_ were measured at the control exposure, and of 6.0 µg/m³ and 3.6 µg/m³, respectively, in the urban background. At the heavy traffic site, median PM_10_ concentrations of 10.2 µg/m³ and PM_2.5_ concentrations of 5.6 µg/m³ were observed, whereas biomass combustion showed the highest values of 13.3 µg/m³ for PM_10_ and 5.9 µg/m³ for PM_2.5_. The highest concentration of NO_2_ (14.4 ppb) and the lowest concentration of O_3_ (17.0 ppb) were measured at the heavy traffic site, while concentrations were similar at other sites. Some of the concentrations of particles and gaseous air pollutants were highly correlated with each other, which was true for UFP and LDSA (Spearman *r* = 0.877, *p* < 0.001), for PM_10_ and PM_2.5_ (Spearman *r* = 0.870, *p* < 0.001), and for O_3_ and NO_2_ (Spearman *r* = -0.565, *p* < 0.001).

**Table 2:** Pollutant concentrations by exposure site based on the median values over the 75 min exposure periods. Comparisons between exposure sites were performed with the nonparametric Kruskal-Wallis test, with *post hoc*-comparisons according to the Benjamini-Hochberg method; the P values given refer to the comparison of the four study sites. ^1^Control exposure vs. Urban background *p* < 0.05, ^2^Control exposure vs. Heavy traffic *p* < 0.05, ^3^Control exposure vs. Biomass combustion *p* < 0.05, ^4^Urban background vs. Heavy traffic *p* < 0.05, ^5^Urban background vs. Biomass combustion *p* < 0.05, ^6^ Heavy traffic vs. Biomass combustion *p* < 0.05. UFP = ultrafine particles, PM = particulate matter, LDSA = lung density surface area, NO_2_ = nitrogen dioxide, O_3_ = ozone, BC = black carbon

Pollutant	Control exposure	Urban background	Heavy traffic	Biomass combustion	*P* value
	**Median (1st Quartile; 3rd Quartile)**				
UFP [#/cm³] ^1 2 3 4 5^	3,162 (2,635; 4,242)	6,497 (4,297; 7,359)	15,932 (10,934; 20,173)	24,170 (13,465; 35,061)	< 0.001
LDSA [µm²/cm³] ^2 3 4 5 6^	12.1 (8.8; 14.8)	14.4 (10.8; 16.9)	26.5 (17.3; 32.5)	53.0 (39.9; 98.8)	< 0.001
PM_2.5_ [µg/m³] ^4 5^	4.7 (2.9; 7.3)	3.6 (2.2; 5.3)	5.6 (4.4; 6.9)	5.9 (4.0; 8.3)	0.009
PM_10_ [µg/m³] ^4 5^	9.6 (5.7; 15.4)	6.0 (4.8; 8.3)	10.2 (7.2; 17.8)	13.3 (8.3; 18.1)	0.001
NO_2_ [ppb] ^2 6^	5.5 (1.3; 12.6)	7.7 (2.1; 22.6)	14.4 (5.7; 27.8)	7.8 (1.8; 11.9)	0.015
O_3_ [ppb] ^2 4 6^	29.5 (12.6; 41.9)	28.4 (11.4; 33.3)	17.0 (5.4; 27.7)	26.2 (18.2; 36.1)	0.015
Ratio UFP / PM_2.5_^1 2 3 5^	654 (449; 1,056)	1,713 (985; 3,742)	2,576 (1,827; 4,308)	4,308 (2,214; 5,985)	< 0.001
Ratio UFP / PM_10_^1 2 3 5^	372 (234; 557)	999 (494; 1536)	1,317 (966; 1913)	1,922 (1122; 2715)	< 0.001
BC [ng/m³] ^2 3 4 5 6^	331.5 (29.7; 520.9)	464.6 (258.1; 632.8)	1,933.8 (1,435.8; 2,624.3)	4,570.3 (3,271.8; 8,920.2)	< 0.001
Temperature [^o^C]	17.9 (11.7; 23.2)	20.4 (14.2; 23.8)	21.8 (17.5; 25.8)	20.5 (18.9; 22.0)	0.246
Absolute humidity [g/m^3^]	9.4 (7.6; 11.2)	8.9 (7.7; 10.7)	8.9 (7.3; 10.1)	9.2 (7.8; 10.4)	0.852

### Symptoms

Exposures did not significantly induce irritative symptoms from the lower or upper respiratory tract, nose or skin. However, headache was induced. Compared to control exposure and urban background, the change in headache was significantly higher at the heavy traffic site and with biomass combustion (*p* < 0.001 each; Table 3). Burning, itchy and tired eyes were averaged into a summary score of eye symptoms, the change of which was significantly (*p* < 0.001) higher with biomass combustion compared to the other sites. For well-being, participants indicated high levels with control exposure and urban background, and a low level of impairment at these two sites. For the heavy traffic site and biomass combustion exposure, the converse was observed. In addition, participants reported smell at all locations, with high intensities for biomass combustion and heavy traffic sites, and low intensities for control exposure and urban background. They also felt bothered by the odor at the heavy traffic site and with biomass combustion.

**Table 3:** Changes of symptoms (headache, eye symptoms; symptom score post-exposure minus pre-exposure) by exposure sites. For well-being, perceived impairment of well-being, smell, and annoying odor post-exposure scores are reported. All symptoms were assessed on 0–100 mm visual analog scales. P values refer to the comparison of the four exposure sites using the Friedman non-parametric test, the P values given referring to the comparison of the four study sites, with pairwise *post hoc*-comparisons according to the Wilcoxon matched-pairs signed-ranks test and the Benjamini-Hochberg method of correction for multiple comparisons. ^2^Control exposure vs. Heavy traffic *p* < 0.05, ^3^Control exposure vs. Biomass combustion *p* < 0.05, ^4^Urban background vs. Heavy traffic *p* < 0.05, ^5^Urban background vs. Biomass combustion *p* < 0.05. ^6^Heavy traffic vs. Biomass combustion *p* < 0.05. Eye symptoms are expressed as mean values of the single symptoms burning eyes, itchy eyes, tired eyes

	Control exposure	Urban background	Heavy traffic	Biomass combustion	*P* value
Δ Headache ^2 3 4 5^	0 (-0.3; 0)	0 (0; 3)	9 (0; 16)	7 (0; 15)	< 0.001
Δ Eye symptoms ^2 3 5^	0 (-3; 0)	0 (-1; 1)	0 (-0.3; 5)	4 (0; 9)	< 0.001
During exposures					
Well-being ^2 3 4 5 6^	100 (78; 100)	80 (74; 91)	47 (35; 65)	69 (56; 85)	< 0.001
Perceived impairment of well-being ^2 3 4 5^	0 (0; 0)	0 (0; 5)	47 (18; 59)	31 (0; 57)	< 0.001
Smell ^3 4 5 6^	14 (0; 53)	4 (0; 15)	42 (20; 69)	88 (75; 100)	< 0.001
Annoying odor ^2 3 4 5^	0 (0; 0)	0 (0; 0)	45 (8; 61)	45 (27; 67)	< 0.001

### Functional parameters

As indicated in Table 4, a number of outcome measures showed statistically significant changes when comparing post- versus pre-exposure values for each or the four exposures.

**Table 4:** Relative difference of post-exposure vs. pre-exposure values (Δ%) of lung function and cardiovascular outcomes. Three types of statistical comparisons are indicated. First, comparisons of pre-exposure values were performed with the Friedman nonparametric test; the p values given in the respective second last column refer to these comparisons. Secondly, the same was done by comparing the percent changes (Δ%) of outcome measures between exposures, and the result is given in the last column. Thirdly, in order to assess whether there were significant changes within each exposure, comparisons between the values pre and post-exposure were performed using the Wilcoxon matched-pairs signed-ranks test; their results are indicated as *p < 0.05, **p < 0.01, ***p < 0.001 at each of the parentheses showing the quartiles. FEV_1_ = forced expiratory volume in 1 s, FVC = forced vital capacity, DLCO = lung transfer factor for carbon monoxide, DLNO = lung transfer factor for nitric oxide, KCO = carbon monoxide transfer coefficient, KNO = nitric oxide transfer coefficient, AV = alveolar volume, FeNO = fractional concentration of exhaled nitric oxide, BP = blood pressure, HR = heart rate, RHI = reactive hyperemia index

Outcome	Control exposure	Urban background	Heavy traffic	Biomass combustion	*P* value for pre-exposure values	*P* value for Δ% between exposures
	**Median** **(1st Quartile; 3rd Quartile)**					
Δ% FEV_1_	0.69 (-1.15; 2.48)	1.45 (0.22; 3.07)***	0.74 (0.00; 1.95)*	1.60 (-0.71; 3.43)*	0.585	0.351
Δ% FVC	-0.07 (-1.91; 1.16)	0.00 (-2.55; 1.66)	-1.35 (-2.92; 1.04)	-0.63 (-2.10; 1.92)	0.580	0.606
Δ% FEV_1_ / FVC	0.66 (-1.21; 2.36)	2.22 (-0.27; 4.09)***	1.72 (0.71; 3.27)**	1.77 (-0.67; 3.62)**	0.461	0.565
Δ% DLCO	-4.14 (-6.89; 0.33)**	-4.81 (-9.90; 0.00)**	-3.69 (-7.07; -1.04)**	-7.11 (-9.10; -1.49)***	0.256	0.158
Δ% DLNO	-2.07 (-4.81; 2.35)	-5.08 (-6.47; -0.48)**	-3.88 (-5.76; -0.00)**	-5.51 (-7.50; -2.13)***	0.616	0.155
Δ% KCO	-2.98 (-5.32; -0.57)***	-3.18 (-6.64; -1.07)***	-1.29 (-5.25; 1.19)	-4.28 (-5.61; 1.31)**	0.149	0.567
Δ% KNO	-1.32 (-4.61; -0.03)**	-3.30 (-5.64; -0.67)***	-3.05 (-5.76; 0.81)*	-3.81 (-5.62; -0.84)***	0.738	0.611
Δ% AV	0.17 (-1.97; 2.73)	-0.36 (-2.61; 1.94)	-0.38 (-3.42; 0.86)	-1.70 (-2.47; -0.17)*	0.192	0.229
Δ% FeNO	6.00 (-7.44; 28.61)	0.00 (-21.63; 17.78)	0.00 (-13.82; 21.73)	5.20 (-12.32; 26.23)	0.827	0.499
Δ% Systolic BP	-6.53 (-10.06; -3.22)***	-3.74 (-8.19; 0.74)*	-5.01 (-9.83; 0.63)**	-3.88 (-8.17; 4.93)	0.103	0.277
Δ% Diastolic BP	-1.85 (-8.35; 3.23)	-4.32 (-8.26; 2.65)*	-1.90 (-6.17; 1.52)	-1.33 (-5.18; 1.73)	0.163	0.626
Δ% HR	0.00 (-12.55; 10.05)	-3.36 (-11.54; 5.48)	-4.33 (-11.81; 8.19)	-8.70 (-18.93; 1.92)**	0.231	0.275
Δ% RHI	12.89 (-1.07; 36.40)*	14.80 (-3.82; 41.15)*	18.27 (5.61; 46.62)*	16.91 (-1.63; 47.16)**	0.143	0.909

### Comparison of baseline values and percent changes between study sites

In the next step, we assessed, whether baseline values prior to exposures had been comparable and whether the percent changes differed between the four study sites. None of the pre-exposure values of lung function and cardiovascular parameters showed statistically significant differences across exposure sites (Table 4). Similarly, none of the Δ% changes of outcome measures showed statistically significant differences when comparing the four study sites (Table 4).

### Results of regression analyses using the individual exposure levels of air pollutants

**Fig. 2:**
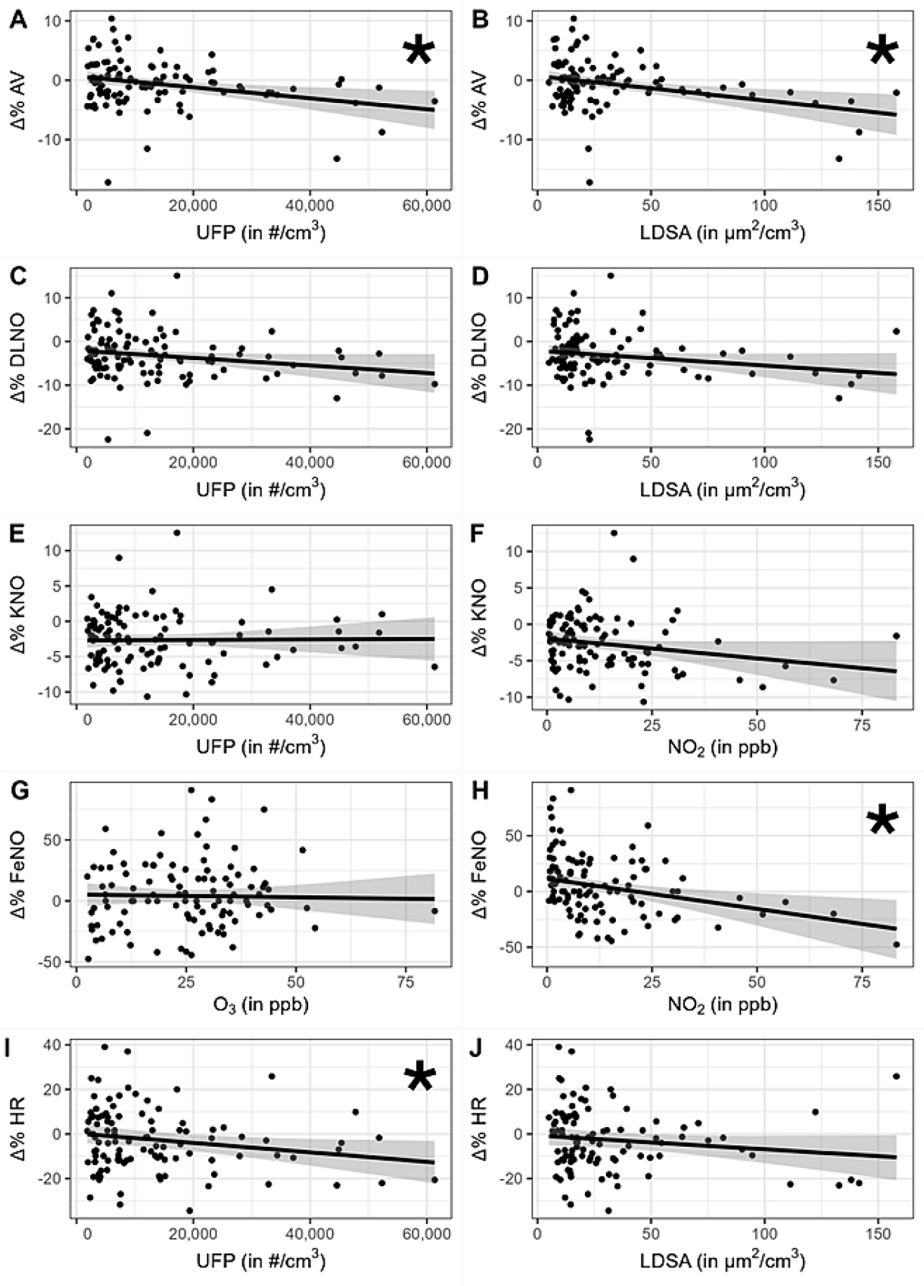
Effect estimates of pollutant concentrations during exposure on the relative difference of post-exposure vs. pre-exposure measurement (Δ%) of outcome variables with linear regression line and 95% interval estimate (grey areas). Black dots represent the measured values. *Interval estimates that exclude 0 (for details see Table 5) are marked with an asterisk. UFP = ultrafine particles, LDSA = lung density surface area, NO_2_ = nitrogen dioxide, O_3_ = ozone, AV = alveolar volume, DLNO = lung transfer factor (diffusing capacity) for nitric oxide, KNO = transfer coefficient for nitric oxide, FeNO = fractional concentration of exhaled nitric oxide, HR = heart rate

Regression analyses were also performed using the percent changes post- versus pre-exposure. There were significant associations of AV with both, UFP and LDSA. Compared to pre-exposure values, post-exposure values of AV decreased with higher UFP numbers (-0.92% change per 10,000/cm³ UFP; 95% CI: -1.57 to -0.28) as well as LDSA levels (-0.41% change per 10 μm/cm³ LDSA; 95% CI: -0.67 to -0.15; Table 5; Fig. 2). Both estimates had been adjusted for PM_2.5_ and NO_2_ levels that were carried in the regression analyses besides temperature and absolute humidity.

**Table 5:** Estimation of the effect of environmental exposures on lung and cardiovascular outcome parameters with regression coefficients and corresponding 95% confidence intervals (CI) in two different models. Interval estimates not containing zero are marked in boldface. The UFP model comprised UFP, NO_2_ and PM_2.5_, temperature and absolute humidity, a global intercept and participant-specific intercepts. The second model was similar but with UFP replaced by LDSA. FEV_1_ = forced expiratory volume in 1 s, FVC = forced vital capacity, DLCO = lung transfer factor for carbon monoxide, DLNO = lung transfer factor for nitric oxide, KCO = carbon monoxide transfer coefficient, KNO = nitric oxide transfer coefficient, AV = alveolar volume, FeNO = fractional concentration of exhaled nitric oxide, BP = blood pressure, HR = heart rate, RHI = reactive hyperemia index, UFP = ultrafine particles, NO_2_ = nitrogen dioxide, PM = particulate matter, LDSA = lung density surface area

Outcome	UFP Model			LDSA Model		
	UFP	NO_2_	PM_2.5_	LDSA	NO_2_	PM_2.5_
	Estimate [95% CI]			Estimate [95% CI]		
∆% Post vs. Pre	Per 10,000 #/cm³	Per 10 ppb	Per 10 µg/m³	Per 10 µg/cm³	Per 10 ppb	Per 10 µg/m³
FEV_1_	0.11 [-0.26 to 0.46]	0.02 [-0.33 to 0.37]	-0.33 [-1.29 to 0.61]	0.07 [-0.07 to 0.21]	0.04 [-0.29 to 0.38]	-0.38 [-1.35 to 0.60]
FVC	0.09 [-0.39 to 0.57]	-0.10 [-0.57 to 0.37]	-0.67 [-1.94 to 0.64]	0.03 [-0.15 to 0.23]	-0.08 [-0.53 to 0.36]	-0.64 [-1.91 to 0.62]
FEV_1_ / FVC	0.00 [-0.47 to 0.46]	0.17 [-0.27 to 0.61]	0.31 [-0.92 to 1.54]	0.03 [-0.16 to 0.21]	0.17 [-0.27 to 0.60]	0.26 [-1.01 to 1.50]
DLCO	-0.73 [-1.71 to 0.29]	0.37 [-0.58 to 1.27]	0.68 [-1.93 to 3.28]	-0.29 [-0.70 to 0.12]	0.22 [-0.69 to 1.13]	0.72 [-1.90 to 3.36]
DLNO	-0.86 [-1.77 to 0.05]	-0.39 [-1.22 to 0.42]	0.89 [-1.46 to 3.16]	-0.34 [-0.70 to 0.03]	-0.56 [-1.35 to 0.25]	0.90 [-1.46 to 3.31]
KCO	0.18 [-0.71 to 1.10]	0.15 [-0.73 to 1.04]	-0.07 [-2.43 to 2.26]	0.11 [-0.25 to 0.48]	0.20 [-0.62 to 1.05]	-0.15 [-2.56 to 2.19]
KNO	0.03 [-0.59 to 0.66]	-0.54 [-1.11 to 0.05]	0.34 [-1.31 to 1.98]	0.07 [-0.19 to 0.32]	-0.54 [-1.10 to 0.03]	0.21 [-1.41 to 1.86]
AV	**-0.92** **[-1.57 to -0.28]**	0.15 [-0.45 to 0.74]	0.61 [-1.12 to 2.29]	**-0.41** **[-0.67 to -0.15]**	-0.03 [-0.62 to 0.57]	0.80 [-1.00 to 2.53]
FeNO	1.12 [-2.46 to 4.85]	**-5.40** **[-9.15 to -1.78]**	-1.66 [-9.64 to 5.51]	0.76 [-0.79 to 2.37]	**-5.19** **[-8.67 to -1.74]**	-1.91 [-10.14 to 5.31]
Systolic BP	1.24 [-0.02 to 2.50]	0.24 [-0.93 to 1.41]	-1.25 [-4.56 to 1.95]	0.45 [-0.05 to 0.95]	0.49 [-0.68 to 1.68]	-1.28 [-4.50 to 1.97]
Diastolic BP	0.03 [-1.18 to 1.24]	0.06 [-1.04 to 1.14]	0.13 [-2.87 to 3.24]	-0.02 [-0.50 to 0.45]	0.07 [-1.04 to 1.13]	0.22 [-2.93 to 3.23]
HR	**-2.12** **[-4.01 to -0.25]**	-0.82 [-2.72 to 1.10]	1.35 [-3.14 to 5.98]	-0.62 [-1.38 to 0.13]	-1.23 [-3.12 to 0.64]	1.09 [-3.41 to 5.84]
RHI	-0.18 [-5.10 to 4.89]	2.70 [-1.81 to 7.33]	2.44 [-6.10 to 12.90]	0.00 [-2.13 to 2.18]	2.64 [-1.85 to 7.57]	2.38 [-6.27 to 13.77]

Compared to pre-exposure values, also post-exposure FeNO levels were associated with NO_2_. They decreased with higher NO_2_ concentrations in the model comprising PM_2.5_ and UFP as predictor (change by -5.40% per 10 ppb NO_2_; 95% CI: -9.15 to -1.78), as well as in the model comprising PM_2.5_ and LDSA as predictor (change by -5.19% per 10 ppb NO_2_; 95% CI: -8.67 to -1.74).

The percent reduction in heart rate post- versus pre-exposure was greater with higher UFP numbers (change by -2.12% per 10,000/cm³ UFP; 95% CI: -4.01 to -0.25) but was not dependent on LDSA values. Finally, the relative decrease in systolic blood pressure was less pronounced with higher levels of UFP (change by 1.24% per 10,000/cm³ UFP; 95% CI: -0.02 to 2.50) or values of LDSA (0.45% per 10 μm/cm³ LDSA; 95% CI: -0.05 to 0.95); these results were not statistically significant, but the limits of confidence intervals very close to zero. As a consequence, since post-exposure systolic blood pressure measurements were generally lower than pre-exposure values (Table 4), individuals exposed to the highest measured values of UFP or LDSA were estimated to approach the pre-exposure levels.

There were no associations for spirometric parameters (FEV_1_ and FVC), carbon monoxide diffusing capacities (DLCO and KCO), diastolic blood pressure, and RHI, but tendencies for associations of DLNO with UFP and LDSA, as well as of KNO with NO_2_ in both, the UFP and the LDSA model (Table 5).

**Fig. 3:**
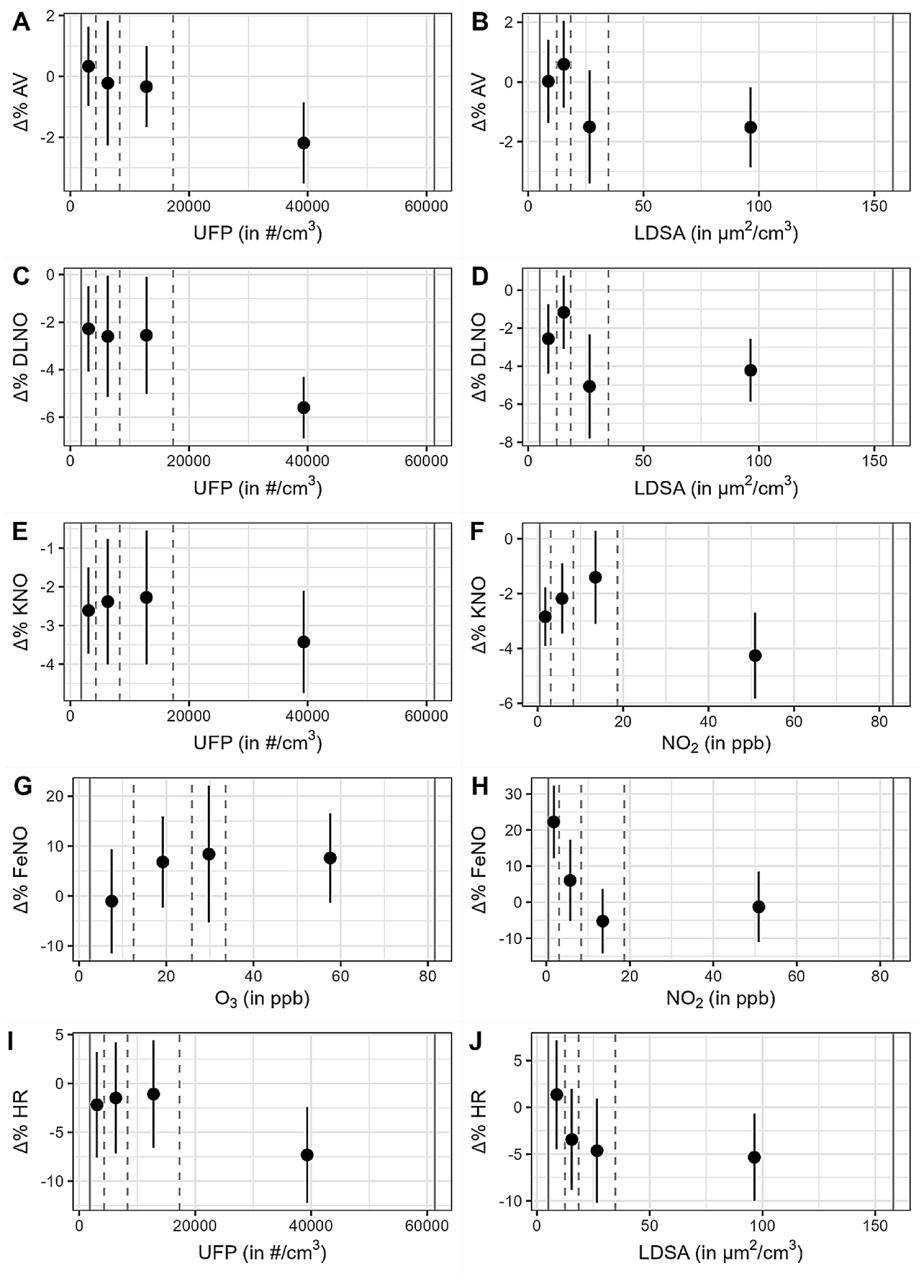
Mean (with confidence interval) of relative difference of post-exposure vs. pre-exposure measurement (Δ%) by quartiles of pollutant concentration. Quartiles are independent of exposure site. The vertical dashed grey lines represent the quartiles, the vertical solid grey lines represent minimum and maximum pollutant concentrations. UFP = ultrafine particles, LDSA = lung density surface area, NO_2_ = nitrogen dioxide, O_3_ = ozone, AV = alveolar volume, DLNO = lung transfer factor for nitric oxide, KNO = nitric oxide transfer coefficient, FeNO = fractional concentration of exhaled nitric oxide, HR = heart rate. The plots are derived from the data points shown in Fig. 2 by categorization of pollutant concentrations into quartiles. Please note the much smaller scale compared to Fig. 2

In additional sensitivity analyses, further adjustment for sex had no influence on any effect estimates. Moreover, the analyses using triple sets of predictors showed no variance inflation compared to analyses not including the covariates but only one air pollutant. Additional inclusion of PM_10_ and O_3_ in the triple models showed the expected multicollinearity regarding the effect estimates of PM_2.5_ and NO_2_, however with little influence on UFP and LDSA estimates. In addition, in all Bayesian analyses the samples of regression coefficients showed low correlation, further indicating negligible multicollinearity effects. To achieve a better impression of the mean changes and their relationship to pollutant levels, quartiles of UFP, LDSA, NO_2_ and ozone levels were computed, and the mean percent changes of AV, DLNO, KNO, heart rate and FeNO together with their 95% confidence intervals were plotted as a function of these quartiles, as shown in Fig. 3. These plots represent the average changes of the data shown in Fig. 2 but categorized into exposure quartiles as presented in Table 2. For interpretation please note the different scales.

## Discussion

The present study followed a semi-experimental design involving realistic exposures to air pollutants at four sites that had been chosen to show systematic differences in their levels of ultrafine particles (UFP), fine particles (PM_2.5_) and gaseous air pollutants (NO_2_, ozone). It included young, healthy, non-allergic individuals, each of whom performed 75-min walks with intermittent light exercise at each of the sites. We thereby combined systematic differences in the levels of air pollutants with a cross-over design in order to maximize statistical power. We also utilized sensitive, non-invasive lung function assessments including lung diffusing capacity for carbon monoxide and nitrogen oxide. On average, the intended exposure conditions were met, but with large overlap of pollutant levels. This was probably the main reason why outcomes did not show significant differences between exposure sites. Anticipating this possibility, we had measured pollutant levels during each exposure allowing for regression analyses based on the actual levels of air pollutants.

These analyses revealed that alveolar volume (AV) was slightly reduced with increasing values of UFP or lung-deposited surface area (LDSA). There was a similar tendency for DLNO, which could be explained by the fact that DLNO depends on the available lung volume stronger than DLCO [12]. These results could be a consequence of a peripheral deposition of UFP and, as one of various possibilities, subsequent induction of peripheral micro-atelectasis, an effect that previously had been postulated for a different type of exposure [12]. It should be emphasized that at present this interpretation is speculative and needs to be substantiated in future studies.

We used DLNO as a tool for measuring gas transport capacity independent from pulmonary capillary blood volume, i.e., the hemoglobin content of the lung. This is possible due to the extremely high affinity of NO for hemoglobin, which renders DLNO practically independent from capillary blood volume. In contrast, the commonly used DLCO is heavily dependent on the hemoglobin content of the lung, due to the lower affinity of CO compared to NO, and therefore reflects the effects of both, capillary blood volume and gas transport to erythrocytes. If additional changes in blood volume would have occurred, these would have resulted in a tendency for greater changes in DLCO compared to DLNO, as previously observed [12]. If there are no such systematic changes, variability of DLCO seems to be larger than that of DLNO, due to the additional factor of blood volume, and DLNO which depends only on gas transport shows more clear changes than DLCO, particularly, if changes are tiny. The observation that DLCO did not change significantly, therefore suggests that pulmonary capillary blood volume was not affected by UFP exposure.

The level of exhaled fractional nitric oxide (FeNO) was slightly increased after all exposures, but this increase was lower with higher levels of ambient air NO_2_. This might be interpreted as an oxidative effect within the central airways. In addition, heart rate declined after all exposures, but this reduction was strongest with high UFP levels, and this effect was statistically significant. Taken together, the observations demonstrate that even in healthy young individuals, short-term exposure to UFP induced a small reduction in the lung volume accessible to gas diffusion, and that ambient air NO_2_ reduced the level of endogenous exhaled nitric oxide.

In contrast to the results of regression analyses, there were no significant differences when comparing the outcomes among the four sites. Besides the overlap of exposure levels, another reason could have been combined effects of air pollutants. We did not address this possibility as it seemed out of reach to disentangle such effects in any reliable manner with the number of experiments given. Therefore, the regression analyses included multiple predictors but no statistical interaction terms.

LDSA is a marker of the potential of particles to interact with the lung via their surface area. Correspondingly to its correlation with UFP, higher values of LDSA were also associated with a reduction in AV. LDSA and UFP showed a similar association with AV, while heart rate was only related to UFP not to LDSA. This suggests that effects from the deposition of particles, due to their number and/or surface area, led to a local peripheral airways response inducing the reduction of lung volume, while the change of heart rate primarily involved particle number but not particle surface. One might speculate that this points toward particle translocation, but again this speculation needs further investigation, although it might be difficult to perform experiments in human subjects. PM_2.5_ and PM_10_ did not show any associations with the outcomes. These observations underline the health-related potential of ultrafine compared to other particles, but of course do not invalidate the findings of other studies that reported effects of PM. In the case, however, that particles of different size are precisely monitored during exposures, UFP seem to beat PM with regard to effects on alveolar volume and heart rate.

We observed a slight overall increase in FeNO after exposures, and no association with particle levels, in contrast to previous studies [25–27]. However, these studies either comprised longer observation periods, or repeated exposures, or did not include gaseous pollutants in their analyses. Moreover, the relative range of variation of PM levels was not extremely high in our study, compared to that of UFP. Our finding of a slight increase in FeNO might involve circadian effects or, in our view more likely, effects elicited by the light exercise. Importantly, the increase was lower with higher levels of NO_2_. This would be compatible with an oxidant effect of NO_2_ on the airway mucosa, in which NO is generated, or with alterations of the mucosa impairing the transfer of NO into the lumen. In a previous study in fighter pilots, a reduction of FeNO was observed, and it was concluded that this was due to drying of the mucosa by the air supplied by the flight support system [12]. To elucidate such an effect, we included absolute humidity as statistical predictor, since the absolute water content of inhaled air determines the amount of water vapor to be generated within the airways. The analyses revealed no significant associations with absolute humidity. It should be noted that we studied non-allergic subjects in whom Th2-related inflammatory effects leading to an increase in FeNO seem very unlikely.

NO_2_ in ambient air is known to be inversely correlated with ozone, and this inverse correlation was also apparent in our data. When replacing NO_2_ with ozone in the regression analysis of FeNO, no association was observed, supporting NO_2_ as the active component. Remarkably, the relative reduction in FeNO occurred even at very low NO_2_ levels. The observation that inhaled oxidants may reduce FeNO is well documented for both active [28–30] and passive smoking [31], with several contributing factors potentially involved [32]. It is unlikely that the reduction in FeNO resulted from a decreased bronchial surface area from which endogenous NO is released, as this would require substantial airway obstruction [33, 34]. Neither FVC, nor FEV_1_, nor its ratio to FVC were statistically significantly dependent on the air pollutant levels, in contrast to findings from other studies [35]. Our observations can also be compared to a study in which patients and healthy subjects aged 60 years and older walked for two hours in either a relatively clean or a heavily polluted environment [4]. In the clean area, spirometric indices improved after the walk compared to those measured before, while this improvement did not occur in the polluted area. In our setting, conducted in young, healthy, well-trained individuals, no comparable effect on spirometric values was detected. NO_2_ also showed a tendency to reduce KNO, the volume-adjusted lung transfer coefficient for NO. The common interpretation of this finding would be a slight impairment in diffusion, possibly due to processes occurring at the alveolar surface. If these involved oxidant effects of NO_2_, they would be consistent with its impact on FeNO. However, since only a tendency was observed, this interpretation remains speculative and warrants further investigation in future studies. Additionally, this finding highlights the potential of NO diffusing capacity over the conventional CO diffusing capacity.

Previous exposure studies described the effects of UFP or other air pollutants on vascular function, using for example flow-mediated dilation [36]. We assessed the reactive hyperemia index (RHI), which has been proposed as a surrogate marker for flow-mediated dilation [37, 38]. While adverse effects of particle number concentration on this outcome have been reported in the presence of physical activity [37], we did not observe any associations with air pollutants. It is possible that changes in peripheral vascular function due to low levels of ambient air pollutants in healthy young individuals require more intense and sustained physical exercise as well as longer exposure times, rather than light, intermittent activity. On the other hand, our findings align with results from experimental exposure studies showing that exposure to concentrated ambient fine and ultrafine particles in a typical urban environment or filtered air for two hours did not affect peripheral vascular vasomotor or fibrinolytic function in twelve male patients with stable coronary heart disease and 12 aged-matched healthy volunteers [38]. Heart rate declined in all exposure scenarios except for the control exposure, with a statistically significant decrease observed during biomass combustion. This decline was associated with high levels of UFP although not LDSA exposure. While this might favor the tentative hypothesis of particle translocation and the dominant role of particle number versus that of surface area, we do not have an explanation for the overall decline. Previous studies found associations between increasing ambient air pollution levels and significant reductions in heart rate variability [39]. In addition, a reduction in personal exposure to air pollution by using a face mask has been linked to small but consistent improvements in heart rate variability in patients with coronary heart disease [40]. However, for heart rate per se no associations have been reported.

Moreover, we observed only a tendency for associations between changes in systolic blood pressure and UFP or LDSA. Possibly, blood pressure was too dependent on other conditions, thereby increasing its variability. A previous study on healthy young women exposed to traffic-related air pollution during physical activity found associations between increased diastolic and systolic blood pressure and ozone levels, but not UFP [37]. However, the study involved higher workload and thus ventilation levels than our study. High exposure to traffic-related pollutants was also found to be associated with an increase in diastolic blood pressure, while various air pollutants (BC, PM_10_, PM_2.5_, UFP, NO_x_) were linked to higher systolic blood pressure. Intermittent physical activity reduced systolic blood pressure overall, particularly in environments with low traffic-related air pollution. The results suggested that physical activity could influence the increases in systolic blood pressure, depending on the type of pollutant [41]. The observed blood pressure increases align with data from indoor particle exposure studies, which have also demonstrated an association with particle surface area (LSDA) [42]. Thus, the available data show a heterogeneous picture regarding cardiovascular parameters and air pollution, in which effects depended on the setting and pollutants analyzed. Probably, in our setting with healthy, young subjects the exposure was too low to exert significant effects beyond the tendencies observed.

In contrast to the changes in the objective measures of lung and cardiovascular function, the changes in symptoms did show significant differences between the exposure sites. Significant impairments regarding headaches and eye symptoms occurred with biomass combustion and at the heavy traffic site but it should be noted that scores were small or very small compared to the total VAS range of 0-100. Accordingly, well-being during exposure, its impairment, smell and annoying odor showed much stronger changes at the heavy traffic site and with biomass combustion compared to control and urban background exposure. While noise exposure could be minimized, it was not possible to blind the participants regarding their exposure sites. Thus, the ratings may have been influenced by visual and other impressions. Conversely, the rating of biomass combustion might have been biased, as many subjects associated positive memories with this exposure, and negative emotions with the heavy traffic site.

### Limitations

One limitation of our study was that only young healthy individuals could be included considering the rather demanding design and assessments. In addition, participants could not be blinded to the exposures, with the consequence that effects from the perception of specific environments could not be entirely ruled out. This was apparent for the self-reported well-being at the heavy traffic site and the biomass combustion. Another limitation was the restricted sample size of 26 participants. Nevertheless, we observed statistical associations, likely due to the cross-sectional study design and the assignment of a broad range of exposure levels to each which suggests that the statistical power was adequate to detect relevant effects. It should be considered that particle composition might have differed between the four exposure sites, as well as potentially the levels of other gaseous pollutants that were not measured. While PM was included primarily for the sake of completeness, our main focus was on UFP. We do not claim that PM has no effects, rather, within the context and scope of our study, any potential PM-related effects were likely weaker and/or less consistent than those of UFP. The absence of significant PM-related effects should therefore not be interpreted as contradictory to previous findings regarding PM. Our study was neither designed nor powered to reproduce such effects. Instead, the focus was on UFP, and the findings suggest that, when properly measured, the effects of UFP are stronger and/or more consistent than those of PM. Some variability in weather conditions was unavoidable, but extreme conditions were prevented by terminating exposures in such cases. Due to the demanding assessment schedule, it was also not feasible to monitor participants over an extended period following exposure to investigate potential delayed effects. It should be emphasized that the observed effects were very small and that their interpretation included significant elements of speculation. Lastly, in real-world exposure conditions, which our study aimed to replicate, particle composition is heterogeneous and variable. A more detailed analysis of compositional effects and related characteristics would have required a substantially larger number of participants and exposure sessions than were possible within the framework of this study.

## Conclusion

Using a randomized, cross-over design with four defined outdoor exposure sites characterized by different levels of ultrafine particles (UFP), fine particles and gaseous air pollutants, we studied 26 young healthy individuals in 75-min exposures with intermittent light exercise. The exposures comprised control, urban background, heavy traffic, and biomass combustion sites. When comparing post- and pre-exposure values, there was a reduction in alveolar volume (AV), i.e., the lung volume accessible to gas transport, with high levels of UFP, or, equivalently, lung-deposited surface area (LDSA). This pointed towards effects on peripheral airways in response to UFP deposition. In addition, the concentration of endogenous exhaled nitric oxide (FeNO) that is thought to originate in the central airways was lower with increasing levels of ambient air nitrogen dioxide (NO_2_), possibly reflecting an oxidant effect. Moreover, there was a stronger decline in heart rate with high levels of UFP. The results indicate that typical ambient air exposures encountered in a large city may have a number of measurable, although subtle acute health effects even in healthy young individuals.

## Data Availability

No datasets were generated or analysed during the current study.

## References

[1] Manisalidis I, Stavropoulou E, Stavropoulos A, Bezirtzoglou E. Environmental and health impacts of air pollution: A review. Front Public Health. 2020;8:14. 32154200 10.3389/fpubh.2020.00014 PMC7044178

[2] Newby DE, Mannucci PM, Tell GS, Baccarelli AA, Brook RD, Donaldson K, et al. Expert position paper on air pollution and cardiovascular disease. Eur Heart J. 2015;36(2):83–b93. 25492627 10.1093/eurheartj/ehu458 PMC6279152

[3] Klepczynska Nystrom A, Svartengren M, Grunewald J, Pousette C, Rodin I, Lundin A, et al. Health effects of a subway environment in healthy volunteers. Eur Respir J. 2010;36(2):240–8. 20032018 10.1183/09031936.00099909

[4] Sinharay R, Gong J, Barratt B, Ohman-Strickland P, Ernst S, Kelly FJ, et al. Respiratory and cardiovascular responses to walking down a traffic-polluted road compared with walking in a traffic-free area in participants aged 60 years and older with chronic lung or heart disease and age-matched healthy controls: a randomised, crossover study. Lancet (London England). 2018;391(10118):339–49. 29221643 10.1016/S0140-6736(17)32643-0 PMC5803182

[5] Van Ryswyk K, Anastasopolos AT, Evans G, Sun L, Sabaliauskas K, Kulka R, et al. Metro commuter exposures to particulate air pollution and PM2.5-Associated elements in three Canadian cities: the urban transportation exposure study. Environ Sci Technol. 2017;51(10):5713–20. 28440082 10.1021/acs.est.6b05775

[6] Garcia A, Santa-Helena E, De Falco A, de Paula Ribeiro J, Gioda A, Gioda CR. Toxicological effects of fine particulate matter (PM(2.5)): health risks and associated systemic Injuries-Systematic review. Water Air Soil Pollut. 2023;234(6):346. 37250231 10.1007/s11270-023-06278-9 PMC10208206

[7] Schraufnagel DE. The health effects of ultrafine particles. Exp Mol Med. 2020;52(3):311–7. 32203102 10.1038/s12276-020-0403-3 PMC7156741

[8] Leikauf GD, Kim SH, Jang AS. Mechanisms of ultrafine particle-induced respiratory health effects. Exp Mol Med. 2020;52(3):329–37. 32203100 10.1038/s12276-020-0394-0 PMC7156674

[9] WHO. WHO global air quality guidelines. WHO global air quality guidelines: Particulate matter (PM(25) and PM(10)), ozone, nitrogen dioxide, sulfur dioxide and carbon monoxide. WHO Guidelines Approved by the Guidelines Review Committee. Accessed October 31. 2024. Accessed October 31, 2024: https://www.ncbi.nlm.nih.gov/pubmed/34662007; 2021. 34662007

[10] McCreanor J, Cullinan P, Nieuwenhuijsen MJ, Stewart-Evans J, Malliarou E, Jarup L, et al. Respiratory effects of exposure to diesel traffic in persons with asthma. N Engl J Med. 2007;357(23):2348–58. 18057337 10.1056/NEJMoa071535

[11] Lepistö T, Lintusaari H, Oudin A, Barreira LMF, Niemi JV, Karjalainen P, et al. Particle lung deposited surface area (LDSAal) size distributions in different urban environments and geographical regions: towards Understanding of the PM2.5 dose–response. Environ Int. 2023;180:108224. 37757619 10.1016/j.envint.2023.108224

[12] Bojahr J, Jorres RA, Kronseder A, Weber F, Ledderhos C, Roiu I, et al. Effects of training flights of combat jet pilots on parameters of airway function, diffusing capacity and systemic oxidative stress, and their association with flight parameters. Eur J Med Res. 2024;29(1):100. 38317201 10.1186/s40001-024-01668-z PMC10840181

[13] Gumperlein I, Fischer E, Dietrich-Gumperlein G, Karrasch S, Nowak D, Jorres RA, et al. Acute health effects of desktop 3D printing (fused deposition modeling) using acrylonitrile butadiene styrene and polylactic acid materials: an experimental exposure study in human volunteers. Indoor Air. 2018;28(4):611–23. 29500848 10.1111/ina.12458

[14] Wurzner P, Jorres RA, Karrasch S, Quartucci C, Bose-O’Reilly S, Nowak D, et al. Effect of experimental exposures to 3-D printer emissions on nasal allergen responses and lung diffusing capacity for inhaled carbon monoxide/nitric oxide in subjects with seasonal allergic rhinitis. Indoor Air. 2022;32(11):e13174. 36437663 10.1111/ina.13174

[15] Karrasch S, Simon M, Herbig B, Langner J, Seeger S, Kronseder A, et al. Health effects of laser printer emissions: a controlled exposure study. Indoor Air. 2017;27(4):753–65. 28054389 10.1111/ina.12366

[16] Quanjer PH, Stanojevic S, Cole TJ, Baur X, Hall GL, Culver BH, et al. Multi-ethnic reference values for spirometry for the 3-95-yr age range: the global lung function 2012 equations. Eur Respir J. 2012;40(6):1324–43. 22743675 10.1183/09031936.00080312 PMC3786581

[17] Dweik RA, Boggs PB, Erzurum SC, Irvin CG, Leigh MW, Lundberg JO, et al. An official ATS clinical practice guideline: interpretation of exhaled nitric oxide levels (FENO) for clinical applications. Am J Respir Crit Care Med. 2011;184(5):602–15. 21885636 10.1164/rccm.9120-11ST PMC4408724

[18] Stanojevic S, Graham BL, Cooper BG, Thompson BR, Carter KW, Francis RW, et al. Official ERS technical standards: global lung function initiative reference values for the carbon monoxide transfer factor for Caucasians. Eur Respir J. 2017;50(3):1700010. 28893868 10.1183/13993003.00010-2017

[19] Wasserman L. All of statistics: a concise course in statistical inference. New York: Springer; 2004. xix, 442 p. p.

[20] Carpenter B, Gelman A, Hoffman MD, Lee D, Goodrich B, Betancourt M, et al. Stan: A probabilistic programming Language. J Stat Softw. 2017;76(1):1–32. 36568334 10.18637/jss.v076.i01 PMC9788645

[21] Bürkner P-C. Brms: an R package for bayesian multilevel models using Stan. J Stat Softw. 2017;80(1):1–28.

[22] Computing RFfS. R: A Language and environment for statistical computing 2024 https://www.R-project.org/: R Core Team; accessed September 19, 2024 [.

[23] Betancourt M. Robust Statistical Workflow with RStan. https://www.mc-stanorg/users/documentation/case-studies/rstan_workflowhtml [Internet]. Stan Case Studies 4, 2017; Accessed October 31, 2024.

[24] Cooper BG, Stocks J, Hall GL, Culver B, Steenbruggen I, Carter KW, et al. The global lung function initiative (GLI) network: bringing the world’s respiratory reference values together. Breathe (Sheff). 2017;13(3):e56–64. 28955406 10.1183/20734735.012717 PMC5607614

[25] Bos I, De Boever P, Vanparijs J, Pattyn N, Panis LI, Meeusen R. Subclinical effects of aerobic training in urban environment. Med Sci Sports Exerc. 2013;45(3):439–47. 23073213 10.1249/MSS.0b013e31827767fc

[26] Mirabelli MC, Golan R, Greenwald R, Raysoni AU, Holguin F, Kewada P, et al. Modification of Traffic-related respiratory response by asthma control in a population of Car commuters. Epidemiol (Cambridge Mass). 2015;26(4):546–55. 10.1097/EDE.0000000000000296 PMC4516050 25901844

[27] Strak M, Janssen NA, Godri KJ, Gosens I, Mudway IS, Cassee FR, et al. Respiratory health effects of airborne particulate matter: the role of particle size, composition, and oxidative potential-the RAPTES project. Environ Health Perspect. 2012;120(8):1183–9. 22552951 10.1289/ehp.1104389 PMC3440077

[28] Malinovschi A, Janson C, Holmkvist T, Norback D, Merilainen P, Hogman M. Effect of smoking on exhaled nitric oxide and flow-independent nitric oxide exchange parameters. Eur Respir J. 2006;28(2):339–45. 16641119 10.1183/09031936.06.00113705

[29] Sundy JS, Hauswirth DW, Mervin-Blake S, Fernandez CA, Patch KB, Alexander KM, et al. Smoking is associated with an age-related decline in exhaled nitric oxide. Eur Respir J. 2007;30(6):1074–81. 17928310 10.1183/09031936.00087807

[30] Marini S, Buonanno G, Stabile L, Ficco G. Short-term effects of electronic and tobacco cigarettes on exhaled nitric oxide. Toxicol Appl Pharmacol. 2014;278(1):9–15. 24732441 10.1016/j.taap.2014.04.004

[31] Schilling J, Holzer P, Guggenbach M, Gyurech D, Marathia K, Geroulanos S. Reduced endogenous nitric oxide in the exhaled air of smokers and hypertensives. Eur Respir J. 1994;7(3):467–71. 8013603 10.1183/09031936.94.07030467

[32] Yates DH, Breen H, Thomas PS. Passive smoke inhalation decreases exhaled nitric oxide in normal subjects. Am J Respir Crit Care Med. 2001;164(6):1043–6. 11587994 10.1164/ajrccm.164.6.2005043

[33] Michils A, Akset M, Haccuria A, Perez-Bogerd S, Malinovschi A, Van Muylem A. The impact of airway obstruction on Feno values in asthma patients. J Allergy Clin Immunol Pract. 2024;12(1):111–7. 37634805 10.1016/j.jaip.2023.08.027

[34] Ragnoli B, Radaeli A, Pochetti P, Kette S, Morjaria J, Malerba M. Fractional nitric oxide measurement in exhaled air (FeNO): perspectives in the management of respiratory diseases. Ther Adv Chronic Dis. 2023;14:20406223231190480. 37538344 10.1177/20406223231190480 PMC10395178

[35] Turner A, Brokamp C, Wolfe C, Reponen T, Ryan P. Personal exposure to average weekly ultrafine particles, lung function, and respiratory symptoms in asthmatic and non-asthmatic adolescents. Environ Int. 2021;156:106740. 34237487 10.1016/j.envint.2021.106740 PMC8380734

[36] Liu L, Kauri LM, Mahmud M, Weichenthal S, Cakmak S, Shutt R, et al. Exposure to air pollution near a steel plant and effects on cardiovascular physiology: a randomized crossover study. Int J Hyg Environ Health. 2014;217(2–3):279–86. 23911139 10.1016/j.ijheh.2013.06.007

[37] Weichenthal S, Hatzopoulou M, Goldberg MS. Exposure to traffic-related air pollution during physical activity and acute changes in blood pressure, autonomic and micro-vascular function in women: a cross-over study. Part Fibre Toxicol. 2014;11:70. 25487431 10.1186/s12989-014-0070-4 PMC4276095

[38] Brauner EV, Moller P, Barregard L, Dragsted LO, Glasius M, Wahlin P, et al. Exposure to ambient concentrations of particulate air pollution does not influence vascular function or inflammatory pathways in young healthy individuals. Part Fibre Toxicol. 2008;5:13. 18837984 10.1186/1743-8977-5-13 PMC2579917

[39] Shutt RH, Kauri LM, Weichenthal S, Kumarathasan P, Vincent R, Thomson EM, et al. Exposure to air pollution near a steel plant is associated with reduced heart rate variability: a randomised crossover study. Environ Health: Global Access Sci Source. 2017;16(1):4. 10.1186/s12940-016-0206-0 PMC5273798 28129768

[40] Langrish JP, Li X, Wang S, Lee MM, Barnes GD, Miller MR, et al. Reducing personal exposure to particulate air pollution improves cardiovascular health in patients with coronary heart disease. Environ Health Perspect. 2012;120(3):367–72. 22389220 10.1289/ehp.1103898 PMC3295351

[41] Kubesch N, De Nazelle A, Guerra S, Westerdahl D, Martinez D, Bouso L, et al. Arterial blood pressure responses to short-term exposure to low and high traffic-related air pollution with and without moderate physical activity. Eur J Prev Cardiol. 2015;22(5):548–57. 25326542 10.1177/2047487314555602

[42] Soppa VJ, Schins RPF, Hennig F, Nieuwenhuijsen MJ, Hellack B, Quass U, et al. Arterial blood pressure responses to short-term exposure to fine and ultrafine particles from indoor sources– A randomized sham-controlled exposure study of healthy volunteers. Environ Res. 2017;158:225–32. 28662448 10.1016/j.envres.2017.06.006

